# A Genomic Perspective Across Earth’s Microbiomes Reveals That Genome Size in Archaea and Bacteria Is Linked to Ecosystem Type and Trophic Strategy

**DOI:** 10.3389/fmicb.2021.761869

**Published:** 2022-01-05

**Authors:** Alejandro Rodríguez-Gijón, Julia K. Nuy, Maliheh Mehrshad, Moritz Buck, Frederik Schulz, Tanja Woyke, Sarahi L. Garcia

**Affiliations:** ^1^Department of Ecology, Environment, and Plant Sciences, Science for Life Laboratory, Stockholm University, Stockholm, Sweden; ^2^Department of Aquatic Sciences and Assessment, Swedish University of Agricultural Sciences, Uppsala, Sweden; ^3^DOE Joint Genome Institute, Berkeley, CA, United States

**Keywords:** microbial ecology, genome size, bacteria, archaea, genomics

## Abstract

Our view of genome size in Archaea and Bacteria has remained skewed as the data has been dominated by genomes of microorganisms that have been cultivated under laboratory settings. However, the continuous effort to catalog Earth’s microbiomes, specifically propelled by recent extensive work on uncultivated microorganisms, provides an opportunity to revise our perspective on genome size distribution. We present a meta-analysis that includes 26,101 representative genomes from 3 published genomic databases; metagenomic assembled genomes (MAGs) from GEMs and stratfreshDB, and isolates from GTDB. Aquatic and host-associated microbial genomes present on average the smallest estimated genome sizes (3.1 and 3.0 Mbp, respectively). These are followed by terrestrial microbial genomes (average 3.7 Mbp), and genomes from isolated microorganisms (average 4.3 Mbp). On the one hand, aquatic and host-associated ecosystems present smaller genomes sizes in genera of phyla with genome sizes above 3 Mbp. On the other hand, estimated genome size in phyla with genomes under 3 Mbp showed no difference between ecosystems. Moreover, we observed that when using 95% average nucleotide identity (ANI) as an estimator for genetic units, only 3% of MAGs cluster together with genomes from isolated microorganisms. Although there are potential methodological limitations when assembling and binning MAGs, we found that in genome clusters containing both environmental MAGs and isolate genomes, MAGs were estimated only an average 3.7% smaller than isolate genomes. Even when assembly and binning methods introduce biases, estimated genome size of MAGs and isolates are very similar. Finally, to better understand the ecological drivers of genome size, we discuss on the known and the overlooked factors that influence genome size in different ecosystems, phylogenetic groups, and trophic strategies.

## Introduction

As microbiologists, how do we define what is a small or a big genome? Perhaps, researchers working on model organisms such as *Escherichia coli* with a genome size of ∼5 Mbp (Abram et al., 2021) would define “big” or “small” differently to researchers working on soil-dwelling bacteria with a genome size of 16 Mbp (Garcia et al., 2014). On the lower genome size scale, whereas genome sizes of bacterial endosymbionts of insects may have genomes merely larger than 100 kbp (Moran and Bennett, 2014), the abundant *Prochlorococcus* range between 1.6 and 1.9 Mbp for high-light and low-light ecotypes (Berube et al., 2018). In summary, it is known that genome sizes of Archaea and Bacteria range between 100 kbp and 16 Mbp, but the genome size distribution in nature is still undefined. Therefore, the aim of this review is to provide an overview of the distribution of genome sizes in different ecosystems.

**FIGURE 1:**
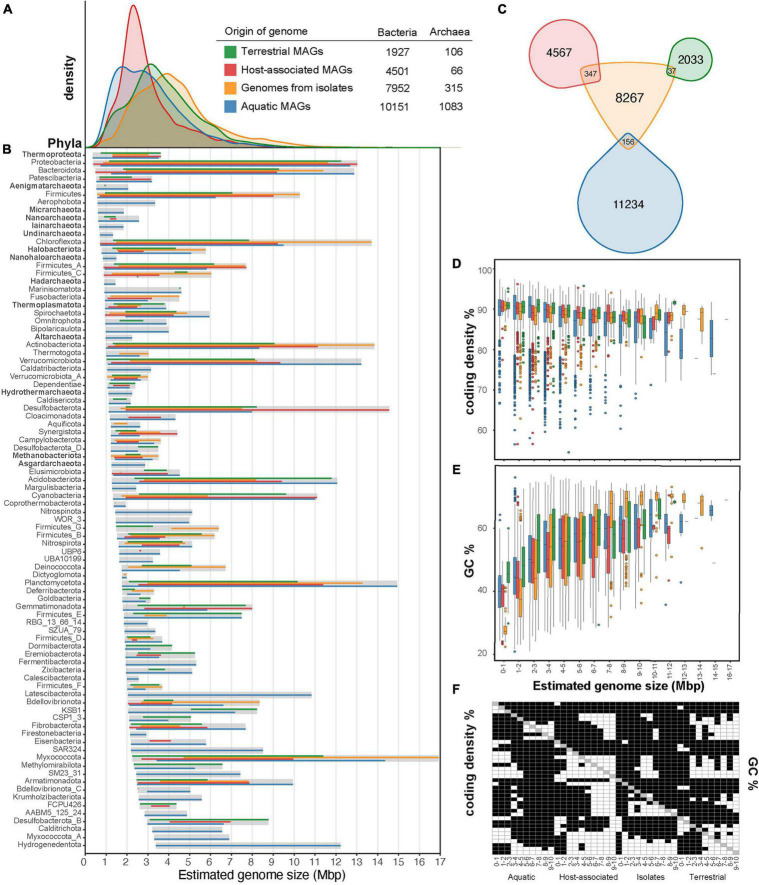
Overview of the genome size distribution across Earth’s microbiomes. Genome size distribution of Archaea and Bacteria **(A)** from different environmental sources and across different archaeal and bacterial phyla **(B)** are shown for a total of 26,101 representative genomes. Isolate genomes were gathered from GTDB (release95) and environmental MAGs were gathered from GEMs (Nayfach et al., 2020) and stratfreshDB (Buck et al., 2021a). We use one representative genome per mOTU (defined by 95% ANI) from the union of GEMs catalog and stratfreshDB in the plots. From the GTDB database, we selected one representative isolate genome per species cluster that was circumscribed based on the ANI (≥95%) and alignment fraction [(AF) > 65%] between genomes (Parks et al., 2020). To construct the figures, we plotted the min-max estimated genome sizes, which were calculated based on the genome assembly size and completeness estimation provided. Venn diagram of the intersection between the representative environmental MAGs and the representative isolate genomes **(C)**. The intersection was calculated using FastANI (Jain et al., 2018) and was determined with a threshold of 95%. The coding density **(D)** and GC content (%) **(E)** are shown for the archaeal and bacterial MAGs across different ecosystem categories and isolates. Pair-wise *t*-test was performed in all variables of **(D,E)** and shown in **(F)**, where white is significant (*p* < 0.05) and black is not significant (*p* > 0.05). In **(B)**, we only included phyla with more than five genomes.

We leveraged recently published databases of archaeal and bacterial metagenome assembled genomes (MAGs) (Nayfach et al., 2020; Buck et al., 2021a) together with isolate genomes to revisit and acquire an updated understanding of the estimated genome size distribution across different ecosystems. In this review, we also discuss the ecological drivers that potentially influence genome sizes. In summary, we found that 76.3% of representative archaeal and bacterial genomes recovered through genome-resolved metagenomics present estimated genome sizes below 4 Mbp. Furthermore, all MAGs from five archaeal phyla (Micrarcheota, Ianarchaeota, Undinarchaeota, Nanohaloarchaeota, and Hadarchaeota) and two bacterial phyla (Coprothermobacterota and Dictyoglomota) were recovered exclusively from aquatic ecosystems and have genome sizes below 2 Mbp (Figures 1A,B).

## Approximation of Genetic Units Using 95% Average Nucleotide Identity and Its Caveats

Species are widely considered congruent genetic and ecological units for sexual eukaryotes (Mallet, 2008; Shapiro and Polz, 2015). However, there is no consensus regarding the concept of species for Archaea and Bacteria. Instead, 95% average nucleotide identity (ANI) has been a widely recognized as a genetic boundary to operationally estimate genetic units or “microbial species” (Konstantinidis and Tiedje, 2005; Varghese et al., 2015; Garcia et al., 2018; Jain et al., 2018). Several genomic and metagenomic studies have verified the existence of sequence discrete genetic units with 95% ANI as boundary (Olm et al., 2020; Rodriguez et al., 2021).

We used the 95% ANI boundary in published datasets (Nayfach et al., 2020; Parks et al., 2020; Buck et al., 2021a) to review and renew our view of the distribution of archaeal and bacterial estimated genome size. To minimize representation biases (Gweon et al., 2017), we included one representative per 95% ANI cluster. The approximation of these clusters will be called mOTUs (metagenomic Operational Taxonomic Units) (Buck et al., 2021b; Garcia et al., 2021). We used only one MAG per mOTU, making the estimated genome size dependent on the MAG assembly and quality. It is known that MAG assembly and binning might discriminate against ribosomal RNAs, transfer RNAs, mobile element functions and genes of unknown function (Nelson et al., 2020; Meziti et al., 2021), and also that completeness could be underestimated for streamlined genomes (Garcia et al., 2015). Despite potential biases introduced by these methodological limitations, we still offer valuable insights on genome size distribution across different environments.

## Extant Genome Size Distribution in the Environment

In this review, we have included ∼64,500 environmental MAGs available via two recently published datasets, stratfreshDB and GEMs. StratfreshDB offers ∼12,000 MAGs (>40% completeness) from 41 stratified lakes and ponds assembled with Megahit (v1.1.13) and binned with Metabat (v2.12.1) (Buck et al., 2021a). GEMs offers ∼52,000 MAGs (>50% completeness) from > 10,000 metagenomes collected from diverse habitats on Earth (Nayfach et al., 2020). GEMs dataset was assembled using metaSPAdes and binned with Metabat (v0.32.5). After dereplication using fastANI and mOTUlizer (Jain et al., 2018; Buck et al., 2021b), our meta-analysis includes 17 834 mOTUs, with one representative MAG each (completeness > 50% and contamination < 5%, assessed with CheckM v1.1.3) (Parks et al., 2015). We complemented these environmental MAGs by adding 8 267 representative genomes (>90% complete) of isolates from GTDB (Parks et al., 2020; Figure 1A). These genomes are marked in the GTDB databases (release 95) to originate from culture collections. After clustering at 95% ANI threshold, 540 mOTUs contained representatives from both environmental MAGs and isolate genomes (Figure 1C). Previous surveys based on 16S rRNA have found that the uncultured microbial fraction could constitute up to 81% of the total microbial diversity on Earth (Lloyd et al., 2018). However, it is known that 16S rRNA underestimate prokaryotic diversity (Rodriguez et al., 2018). Overall, our review shows that 3% of the reconstructed environmental mOTUs are represented among cultured microbes.

Furthermore, using completeness estimates from CheckM, we compared the estimated genome size distribution of all MAGs vs. genomes from isolates. The estimated genome size was calculated by dividing the MAG’s assembly size by CheckM completeness (ranging from 0 to 1). Representative genomes from isolates have an average genome size of 4.3 Mbp which is significantly larger than that of MAGs (*t*-test *p* < 0.0001), both when comparing Archaea and Bacteria combined and separately. To compare estimated genome sizes between MAGs, ecosystem type was used according to the GEMs database. Although the ecosystem classification presented here is coarse and might contain countless niches, it still allowed us to see trends for genome sizes. Estimated genome sizes of aquatic MAGs have an average of 3.1 Mbp, host-associated MAGs average 3.0 Mbp, and terrestrial MAGs average 3.7 Mbp (Figure 1A). For the 540 mOTUs that contained both environmental MAGs and isolate genomes (Figure 1C), we found that MAGs were estimated on average 3.7% smaller than isolate genomes (Supplementary Figure 1). In other words, even when assembly and binning methods introduce biases, estimated genome size of MAGs and isolates are very similar. Overall, this suggests that the bias in metagenome assembly and binning would not account for the genome size difference observed between all isolate representatives and ecosystem MAGs, neither for the differences among ecosystem MAGs.

A reason for the difference in genome size between isolates and genomes reconstructed from metagenomes might be related to the fact that traditional isolation techniques select for rare microorganisms (Shade et al., 2012) and do not capture the entire ecosystem’s diversity (Figure 1C). For example, it is known that classical cultivation techniques with rich media bias the cultivation toward copiotrophic and fast-growing microorganisms (Swan et al., 2013). Cultivation biases our view of nature because it selects against slow growing microorganisms (Imachi et al., 2020), host dependency (Cross et al., 2019), and dormancy (Hoehler and Jorgensen, 2013) among others. In nature there are many microorganisms with very limited metabolic capacity (Figueroa-Gonzalez et al., 2020) that is linked with dependencies and smaller genomes sizes (Morris et al., 2012). Microorganisms in nature have coevolved with other microorganisms and might have specific requirements that are difficult to mimic in batch-culture standard-media isolation techniques (Garcia, 2016). Although there have been many advances on cultivation techniques (Dedysh, 2011; Carini et al., 2013; Henson et al., 2016; Imachi et al., 2020), more innovations to culture the uncultivated microbial majority (Lewis et al., 2020) will enable us to bring more natural abundant representatives to culture.

**FIGURE 2:**
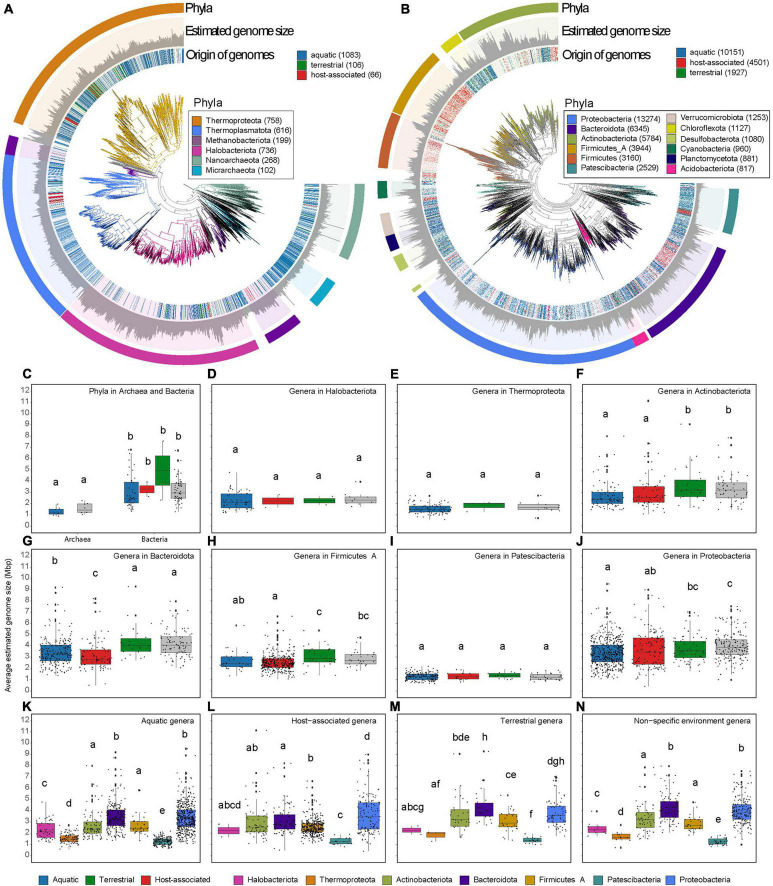
Phylogenetic trees of archaeal **(A)** and bacterial **(B)** representative genomes show variation in genome size between and within phyla. The trees were constructed using GTDB-tk (v 1.5.0) using *de novo* workflow using aligned concatenated set of 122 and 120 single copy marker proteins for Archaea and Bacteria, respectively (Chaumeil et al., 2020). Moreover, in this mode, GTDB-tk adds 1,672 and 30,238 backbone genomes for Archaea and Bacteria, respectively. Tree is visualized in anvi’o (Eren et al., 2021). Estimated genome size is presented in scale from 0 to 6 Mpb or 0 to14 Mbp for Archaea or Bacteria. In the tree, the origin of the environmental genomes is labeled: aquatic, terrestrial and host-associated (same MAGs as Figure 1). Highlighted phyla with more representative genomes are color-coded. Boxplots show the average estimated genome size per phyla within archaeal and bacterial **(C)** domains. The average estimated size per genus within Halobacteriota **(D)**, Thermoproteota **(E)**, Actinobacteriota **(F)**, Bacteroidota **(G)**, Firmicutes A **(H)**, Patescibacteria **(I)**, Proteobacteria **(J)**. The presence of phyla and genera is colored in gray if they contain MAGs from different ecosystem category (non-specific ecosystem). The average estimated size per genus extracted from aquatic ecosystems **(K)**, host-associated ecosystems **(L)**, terrestrial ecosystems **(M)**, or non-specific ecosystems **(N)**. Letters in boxplot panels are the result of non-parametric tests, Wilcoxon **(C)** or Kruskal-Wallis **(D–N)**. Different letters show significant differences *p* < 0.05 (all statistical test with multiple testing were corrected with Benjamini-Hochberg).

Placing archaeal and bacterial genome sizes in phylogenetic trees using GTDB-tk (Figures 2A,B) shows that the distribution of representative genomes and their estimated sizes varies widely between different phyla and within phyla. MAGs assigned to eight phyla in the domain Archaea were reconstructed exclusively from aquatic ecosystems, whereas eight other archaea phyla were reconstructed from multiple ecosystems. There was no significant difference between the genome sizes of aquatic archaea phyla or those from non-specific ecosystem (Figure 2C). However, estimated genome sizes in bacterial phyla were significantly larger than those in archaeal phyla. Moreover, genera from phyla with genome sizes below 3 Mbp, such as Halobacteriota, Thermoproteota, and Patescibacteria, do not show genome size variation in different ecosystems (Figures 2D,E,I). Nevertheless, genera from these smaller genome sizes phyla are significantly smaller than genera with more genome size variation in any ecosystem category (Figures 2K–N). For phyla with genome sizes above 3 Mbp, the genome sizes in aquatic or host-associated genera are significantly smaller than those in terrestrial or non-specific ecosystems (Figures 2F–J). We observe that while the microorganisms’ ecosystem can certainly be linked to genome size, phyla where genome sizes are mostly below 3 Mbp show no variation in estimated genome size across ecosystems.

Clustering microorganisms together by the three ecosystem categories is not optimal since each contains innumerable niches. In each niche, there will be different selective pressures on the genome size. An example is clearly shown in a study (Nayfach and Pollard, 2015) in which it is observed that Archaea and Bacteria sampled from different parts of the human body have differences in genome sizes. Low metadata resolution and clustering of all genomes into three main ecosystem types might be a reason why we see a range of genome sizes in the genera of different ecosystems (Figure 2). With more precise metadata and higher sampling resolution of microhabitats, it might be possible to identify the ecological drivers of genome sizes in the different niches in fine-scale.

## Impact of Ecosystem and Trophic Strategy on Genome Size

Terrestrial ecosystems harbor immense microbial diversity (Delgado-Baquerizo et al., 2018). Yet, the most up-to-date data compilation provided here shows only 2033 MAGs from terrestrial ecosystems (Figure 1C) with an average genome size of 3.7 Mb (Figure 1A). The sub-ecosystems considered in this view are soil and deep subsurface, among others (Supplementary Figure 2). While the terrestrial microorganism’s genome size is the biggest of the three ecosystem categories in this review, they are smaller than expected based on previous metagenomic predictions, which placed the genome size of soil bacteria at 4.74 Mb (Raes et al., 2007). Trends for larger genome sizes in soil have been hypothesized to be related to scarcity and high diversity of nutrients, and a fluctuating environment combined with little penalty for the slow growth rate (Konstantinidis and Tiedje, 2004; Cobo-Simon and Tamames, 2017; Chen et al., 2021). Although terrestrial environments are physically structured, they are generally characterized by two to three orders of magnitude greater variations (in temperature and currents) than marine environments (Steele et al., 2019). *In silico* studies predict that large genome sizes could result from higher environmental variability (Bentkowski et al., 2015). A recent example showed that isolates of terrestrial Cyanobacteria have genomes on the larger size scale (6.0–8.0. Mb) that are enriched in genes involved in regulatory, transport and motility functions (Chen et al., 2021). These functional categories enable them to thrive in a fluctuating environment with high nutrient diversity. Despite these general trends showing larger genome sizes in terrestrial ecosystems, it is worth noting that the diversity captured in the GEMs survey is probably a small fraction of the total terrestrial microbial diversity. It is, for example, also known that streamlined microorganisms such as Patescibacteria (Ortiz et al., 2021) and “*Candidatus* Udaeobacter copiosus” (Verrucomicrobiota) are abundant in soils (Brewer et al., 2016). Therefore, we predict that the view on genome size distribution and microbial diversity in terrestrial ecosystems will become more complete with more sequencing, assembly, binning and novel isolation efforts.

In host-associated microbiomes, genetic drift, deletion biases and low populations sizes drive the reduction of genomes (Li et al., 2021). In these environments, the differing levels of intimacy with their host can influence the evolution of the genome of microorganisms. For example, within the Chlamydiaceae family, some lineages have evolved intracellular associations with eukaryotes (Toft and Andersson, 2010; Collingro et al., 2020). These intracellular Chlamydiaceae have lost many genes that were likely present in their common ancestor that lived in the environment (Dharamshi et al., 2020). Moreover, host-associated bacterial genomes show a variation in size depending on the type of host (plant, animal, etc.) and the type of association they have with the host, such as endosymbiotic, ectobiotic, or epibiotic (Supplementary Table 1). Generally, microorganisms associated with Arthropoda (Tamas et al., 2002), humans (McLean et al., 2020) and other mammals show smaller genomes sizes, whereas protist- and plant-associated bacteria present larger genomes (Levy et al., 2017; Supplementary Figure 2). In fact, *in silico* studies of Alphaproteobacteria show massive genome expansions diversifying plant-associated Rhizobiales and extreme gene losses in the ancestor of the intracellular lineages Rickettsia, Wolbachia, Bartonella, and Brucella that are animal- and human-associated (Boussau et al., 2004). Although host-associated microorganisms are widely known for their reduced genomes, the characteristics of host-associated MAGs show coding densities of ∼91% for genomes below 2 Mbp (Figure 1D).

Small genomes exhibit either strong dependency on other community members or have specific nutrient requirements. Two diverging views on genome reduction have emerged to explain mechanisms of gene loss. On the one hand, genetic drift is more pronounced in species that have a small effective population size, such as host-associated endosymbiotic microorganisms. These microorganisms might thrive because hosts provide energy or nutrients. On the other hand, streamlining is the process of gene loss through selection and it is mainly observed in free-living microorganisms with high effective population sizes (Giovannoni et al., 2014). Some of the most numerically abundant and streamlined microorganisms known to date, such as Pelagibacter (class Alphaproteobacteria) (Giovannoni et al., 2014), *Prochlorococcus* (phylum Cyanobacteria) (Rocap et al., 2003) Thermoproteota (Aylward and Santoro, 2020) and Patescibacteria (Tian et al., 2020), are commonly found in aquatic niches. Paradoxically, even though these microorganisms are free-living, their small genomes increase their nutritional connectivity to other individuals (Giovannoni et al., 2014). Free-living aquatic microorganisms have been used as exemplary streamlining cases in which many have gone through community adaptive selections and gene loss (Morris et al., 2012). Genome reduction can be so intense that microorganisms lose the capacity to biosynthesize essential metabolites and, thus, become auxotrophs. To overcome required nutritional needs, microorganisms thrive in functional cohorts (Mondav et al., 2020). As opposed to prototrophic lifestyle, the auxotrophic lifestyle is reflected by smaller genome sizes (Grote et al., 2012; Garcia et al., 2015; Brewer et al., 2016; Kang et al., 2017; Supplementary Table 1). An opportunity for future studies includes research on auxotrophy prevalences across the entire spectrum of metabolites (amino acids, nucleotides, fatty acids, vitamins, etc.) in different microbial communities and how those auxotrophies are linked with genome size.

In this review, the largest fraction of MAGs is recovered from aquatic environments. The two main sub-ecosystems in our survey are freshwater with MAGs estimated average genome size of 3.2 Mbp significantly different (*p* < 0.0001) from marine genome size distribution with average estimated genome size of 2.9 Mbp. When comparing freshwater and marine environments, the most obvious difference is salinity followed by nutrient concentration. Further exploring the impact of differing levels of salinity on genome size is an interesting research prospect. Additionally, we compared the union of representative freshwater MAGs from both databases (StratfreshDB and GEMs) (Supplementary Figure 3). The difference of mean estimated genome size between the representatives from freshwater GEMs and StratfreshDB is 0.52 Mbp. However, this is because each database captures genetic units that were not found in the other database.

In general, aquatic environments are vertically structured by gradients of light penetration, temperature, oxygen, and nutrient (Supplementary Table 1). Moreover, microorganisms might experience a microscale spatial and nutrient structure due to the presence of heterogeneous particles. These aquatic structures are drivers of the genetic repertoire of aquatic microorganisms. Metagenomic sequencing reported the increase of genome sizes for Archaea and Bacteria with increasing depths (Mende et al., 2017). Temperature may be as important; for example, a study based on twenty-one Thermoproteota and Euryarchaeota fosmids (Euryarchaetoa currently reclassified into Methanobacteriota, Halobacteriota, and Nanohaloarchaeota) showed high rates of gene gains through HGT to adapt to cold and deep marine environments (Brochier-Armanet et al., 2011). It has been observed that light is a relevant driver of genome size in aquatic environments as it decreases with depth. Photosynthetic bacteria such as *Prochlorococcus* spp. are well-differentiated into a high-light adapted ecotype with smaller genome sizes (average 1.6 Mbp) and a low-light-adapted ecotype with a slightly bigger genome size (average 1.9 Mbp) (Berube et al., 2018; Supplementary Table 1). Limitation of nutrients such as nitrogen (Elser et al., 2007) might also be one of the central factors determining genomic properties (Grzymski and Dussaq, 2012). Nitrogen fixation is a complex process that requires a great amount of genes (Franche et al., 2008) and most nitrogen-fixing marine cyanobacteria have the largest genomes in its phylum (Bergman et al., 2013).

Diversity and quantity of nutrients might be two understudied factors that drive ecology and genome size evolution. A recent example shows that polysaccharide xylan triggers microcolonies, whereas monosaccharide xylose promotes solitary growth in Caulobacter (D’Souza et al., 2021). This is a striking example of how nutrient complexity can foster diverse niches for well-studied cells such as Caulobacter with genome size 4 Mbp. To fully understand the link between genome size and nutritional requirements of diverse environmental microorganisms, we need to systematically explore the ∼90% of molecules/metabolites still unknown (Wienhausen et al., 2017; Hawkes et al., 2018; Patriarca et al., 2020). The wide nutrient complexity in the environment might prompt microorganisms to shape their genome. Their genomic content and metabolic potential defines whether they are capable to feed on the available nutrients, forcing them to develop dependencies with other community members in order to acquire energy and metabolic precursors. Metagenomics combined with metabolomics will provide an understanding of the link between genome size evolution of microorganisms and their nutritional and trophic strategy.

## Conclusion

This review offers a broad overview of genome size distribution across three different ecosystem categories, showing that MAGs recovered from aquatic and host-associated ecosystems present smaller estimated genome sizes than those recovered from terrestrial ecosystems. Moreover, genomes obtained from environmental samples present a smaller estimated genome size than obtained by cultivation approaches. We find that the distribution of genome sizes across the phylogenetic tree of Archaea and Bacteria can be linked to the ecosystem type from which the microorganisms’ genomes have been extracted (aquatic, host-associated or terrestrial). Finally, we review the ecological factors that may cause the varying sizes of genomes in different ecosystems. In comparison with the aquatic and host-associated ecosystems, terrestrial ecosystems might harbor microorganisms with bigger estimated genome sizes mainly due to higher fluctuations in this ecosystem. Host-associations might shape genomes sizes differentially based on the type of host and level of intimacy between the microorganisms and the host. Genomes in aquatic ecosystems might be shaped by vertical stratification of abiotic factors such as nutrient distribution, light penetration, and temperature. Moreover, different trophic strategies such as auxotrophies might be connected to smaller genome sizes. We expect that as the microbial ecology field keeps moving forward with sequencing, bioinformatics, chemical analysis, and novel cultivation techniques, we will get a deeper resolution on physicochemical, metabolic, spatial, and biological drivers of archaeal and bacterial genome sizes.

## Author Contributions

SG, AR-G, and JN conceptualized the literature and data review idea. JN, MB, and FS gathered the data. AR-G and JN performed data analysis. SG, AR-G, and MM drafted the first manuscript. All authors did literature searches, contributed to the writing, and editing of the manuscript.

## Conflict of Interest

The authors declare that the research was conducted in the absence of any commercial or financial relationships that could be construed as a potential conflict of interest.

## Publisher’s Note

All claims expressed in this article are solely those of the authors and do not necessarily represent those of their affiliated organizations, or those of the publisher, the editors and the reviewers. Any product that may be evaluated in this article, or claim that may be made by its manufacturer, is not guaranteed or endorsed by the publisher.
