# Supplementary material for: A Genomic Perspective Across Earth’s Microbiomes Reveals That Genome Size in Archaea and Bacteria Is Linked to Ecosystem Type and Trophic Strategy
Source: Front Microbiol. 2022 Jan 5;12:761869. doi: 10.3389/fmicb.2021.761869 (PMC8767057; doi:10.3389/fmicb.2021.761869)
Supplement: Supplementary file 1 [file Data_Sheet_1.docx]

Supplementary Material

# Supplementary Figures and Tables

**Supplementary Figure 1.** Comparison of conspecific MAGs and isolate genomes. In total, 17834 representative MAGs recovered from the environment were clustered with 8267 reference genomes from isolates into mOTUs at 95% ANI. Only 560 MAGs formed clusters with 556 isolate genomes resulting in 540 mOTUs. Each point in the plot represents a MAG/mOTU pair assigned to a single mOTU. The x-axis indicates the estimated genome size of isolates genomes and the y-axis indicates the estimated genome size of MAGs.


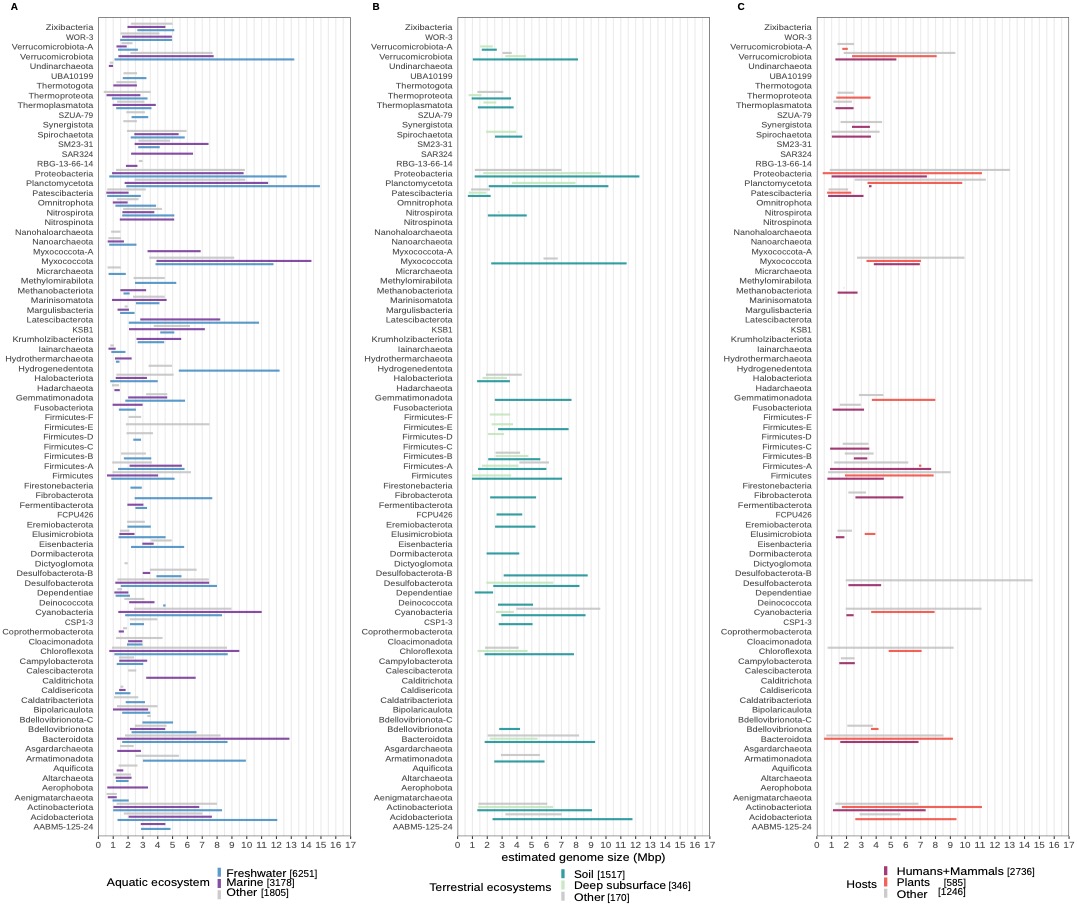


**Supplementary Figure 2.** Distribution of min-max estimated genome sizes per phyla in aquatic [A], terrestrial [B], and host-associated ecosystems [C]. In each panel, the two sub ecosystems are shown from which the most MAGs were recovered, while 'Others' combine MAGs from less represented sub ecosystems


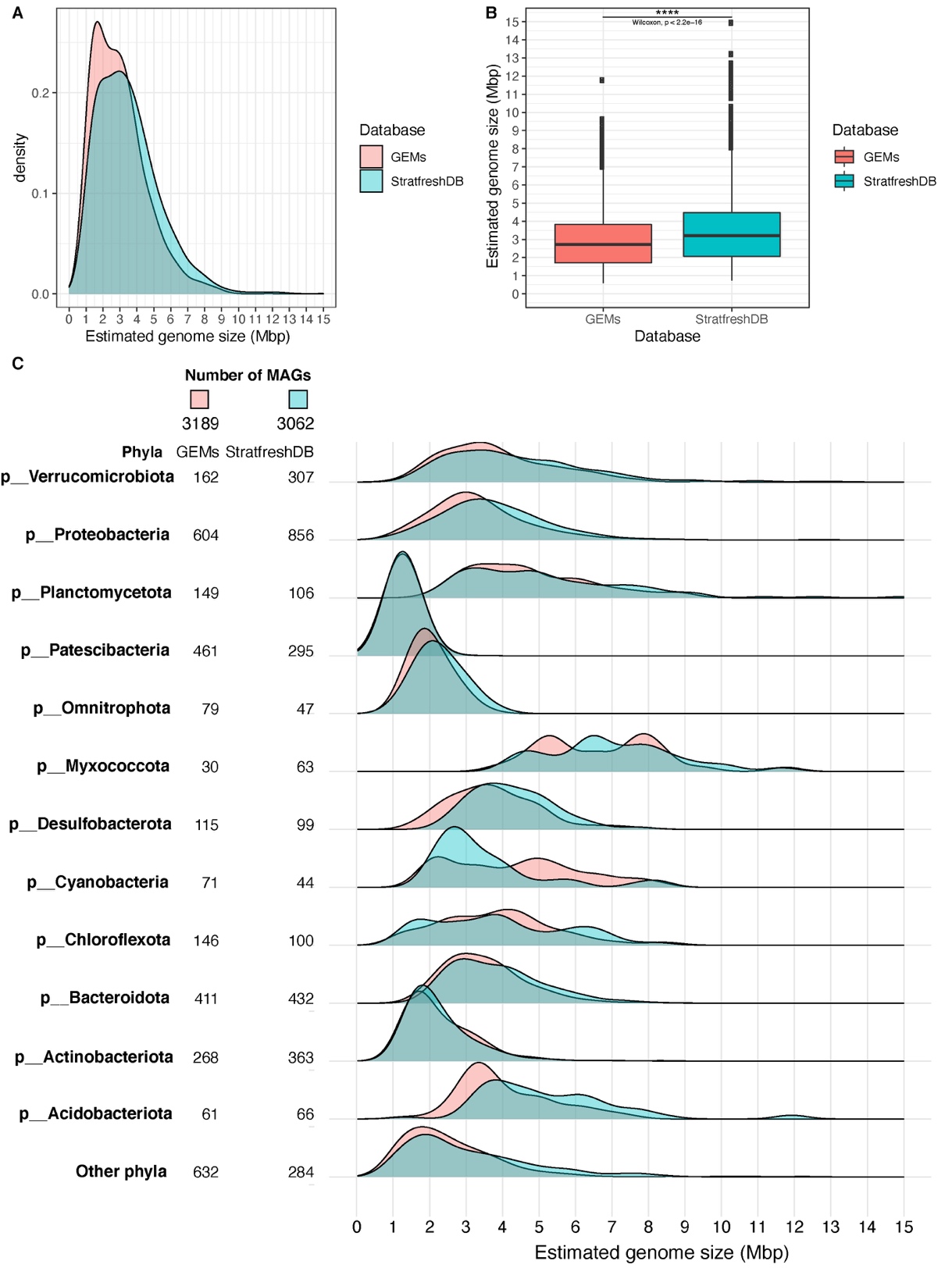


**Supplementary Figure 3.** Overview of the predicted genome size distribution across freshwater biomes in Earth using two databases, GEMs and StratfreshDB. [A] Density plot comparing estimated genome size of GEMs vs StratfreshDB. [B] Boxplot of estimated genome sizes of GEMs vs StratfreshDB. [C] Twelve phyla with highest number of MAGs for both databases (StratfreshDB and GEMs) and density plots of estimated genome sizes per phyla. ‘Other phyla’ combines MAGs from all phyla that are less represented by MAGs in the databases.

**Supplementary Table 1.** Ecological factors and their correlation to genome size.

| **Chemical, physical or biological variable influencing genome size** | | **Taxa** | **References** |
| --- | --- | --- | --- |
| Temperature | *Literature review indicates a negative correlation between genome size and temperature.* | | |
|  | Comparative genomic of genomes of hyperthermophilic microorganisms shows average genome sizes of about 2.3 Mb with very active horizontal gene transfer (HGT) mechanisms | *Thermus thermophilus* (phylum Deinococcota)  *Thermus spp.* | (Blesa et al 2018, Borges and Bergquist 1993) |
|  | Metagenomics suggest that gene gains would have played an important role in adaptation to low temperature and oligotrophic deep marine environments | Thermoproteota and Euryarchaeota (phyla) | (Brochier-Armanet et al 2011) |
|  | Comparative genomics of isolates in one genus indicate larger genomes in colder environments. | *Janthinobacterium* spp. (class Gammaproteobacteria) | (Dieser et al 2019) |
|  | Soil microorganisms show evidence for an inverse relationship between genome size and temperature | Different Archaea and Bacteria | (Sorensen et al 2019) |
|  | Environmental samples indicate that hypersaline environments could increase gene gain via HGT, whereas thermal environments decrease it. | Halobacteria and Thermoproteia (class) | (Rhodes et al 2011) |
| Nutrients | *Diversity and quantity of nutrients are two factors that drive ecology and evolution of genome size. Literature present conflicting results on the effect of these dimensions. Future work should use more sophisticated chemical analytics to characterize quality and quantity of diversity of nutrients to find more predictable correlations.* | | |
|  | Metagenomics indicate dominance of reduced genomes in the Baikal Lake. Small genomes are thought to reflect the extremely oligotrophic conditions of the lake. | Actinobacteria, Bacteroidetes, Cyanobacteria Verrucomicrobia and Thermoproteota (phyla) | (Cabello-Yeves et al 2018) |
|  | Online databases indicate that larger genome-sized species may dominate environments where resources are scarce but diverse. | 70 closely related bacterial genomes | (Konstantinidis and Tiedje 2004) |
|  | Phylogenomics of isolates show gene loss in functions like resource scavenging and energy acquisition when adapting to nutrient-rich environments in algae and corals. | Roseobacter spp. (class Alphaproteobacteria) | (Chu et al 2020) |
|  | Oceanic metagenomic data show positive correlation between nutrient concentration and genome size. | Different bacteria phyla | (Allen et al 2012) |
|  | Metagenomics indicates small genomes in mesopelagic environments are the result of adaptation to energy scarcity. | Some Thermoproteota (phylum) | (Aylward and Santoro 2020) |
|  | Whole-genome shotgun sequencing indicated that deep oligotrophic marine environments are dominated by large genomes with high GC content. | Lactobacillales (phylum Firmicutes) | (Makarova et al 2006) |
|  | Oceanic metagenomic samples suggest that deeper areas with more nitrate and phosphate as nutrients are dominated by large genomes and high GC content. | Bacteria (SAR11, *Prochlorococcus* spp., *Roseobacter* spp., etc.) and Archaea (phyla Thermoproteota and Euryarchaeota) | (Mende et al 2017) |
| Oxygen | *Oxygen promotes bigger genome sizes* | | |
|  | Aerobic microorganisms show larger genome sizes than anaerobes across different environments | Diverse archaeal and bacterial taxa | (Nielsen et al 2021) |
| Trophic strategy | *Some trophic strategies (such as prototrophy, phototrophy, nitrogen fixation) require more genes hence a larger genome size* | | |
|  | Nitrogen-fixation in symbiotic microbes show genome sizes above 7 Mbp. | *Agromonas oligotrophica* and *Bradyrhizobium* spp. (phylum Proteobacteria) and Nostoc spp. (phylum Cyanobacteria) | (Bromfield et al 2019, Gagunashvili and Andresson 2018, Okubo et al 2013) |
|  | Marine isolates show bigger genome sizes in copiotrophs than in oligotrophs. | Copiotrophs (family Vibrionaceae and *Photobacterium angustum)* and oligotrophs (family Sphingomonadaceae and *Sphingopyxis alaskensis*) (phylum Proteobacteria) | (Lauro et al 2009) |
|  | In silico studies indicate that larger genome sizes could have access to a wider variety of carbon substrates but with lower efficiency in carbon usage than smaller genome microbes. | Diverse bacterial taxa (phyla Proteobacteria, Actinobacteriota, Acidobacteriota, Firmicutes, Verrucomicrobiota and Planctomycetota) | (Saifuddin et al 2019) |
|  | Auxotrophs have streamlined genomes. | Actinobacteriota, Proteobacteria, Verrucomicrobiota (phyla) | (Brewer et al 2016, Garcia et al 2015, Grote et al 2012, Kang et al 2017) |
|  | Members of cyanobacteria that don’t have photosynthetic capacities have genome sizes between 1.9 and 2.3 Mbp. | Former *Candidatus* Melainabacteria now in the class Vampirovibrionia | (Di Rienzi et al 2013) |
| Light | *In oxygenic phototrophs there is negative correlation between light irradiance and the genome size.* | | |
|  | Genomes of cultures and single cells show high-light-adapted ecotypes with smaller genome sizes and low-light-adapted ecotypes with bigger genomes. | *Prochlorococcus* spp. (phylum Cyanobacteria) | (Berube et al 2018, Dufresne et al 2003, Rocap et al 2003) |
| Particles | *Microorganisms with particle associated lifestyle tend to have larger genome sizes.* | | |
|  | Comparison of metagenomes in coastal ecosystems show larger genome sizes for particle associated microorganisms than free-living. | Metagenomic data | (Smith et al 2013) |
|  | Particle associated microbes have larger genome sizes than free-living bacteria. | Cyanobacteria and Bacteroidetes (phyla) | (Allen et al 2012) |
| Host-association | *Host-associated bacterial genomes show a variation in size depending on the type of host (plant, animal, etc.) and the type of association they have with the host (endosymbiotic, ectobiotic or epibiotic)* | | |
|  | In silico studies indicate massive genome expansions in plant-associated bacteria. | Alphaproteobacteria (class) | (Boussau et al 2004) |
|  | Isolates from sugarcane (*Saccharum* sp.) rhizosphere and endophytic roots and stalks show 26 individual genomes of associated bacteria whose genomes ranged from 3.9 to 7.5 Mbp. | Diverse bacterial taxa (Burkholderiaceae, Rhizobiaceae, Caulobacteraceae, Xanthomonadaceae, etc.) | (de Souza et al 2019) |
|  | Genomic comparison of 3837 bacterial genomes identified thousands of plant-associated gene clusters and found genomes of plant associated microorganisms tended to be larger | Diverse bacterial taxa | (Levy et al 2017) |
|  | Intense genome reduction in isolates of microbes associated with aphids (Arthropoda). | *Buchnera aphidicola* (class Gammaproteobacteria) | (Tamas et al 2002) |
|  | *In vitro* cultures and metagenomic datasets indicate reduced genome sizes in microbes associated with humans and other mammmals | *Salmonella enterica* (class Gammaproteobacteria)  Patescibacteria (phylum) | (McLean et al 2020, Nilsson et al 2005) |
|  | Environmental samples indicate that symbionts and epibionts of other microbes present highly reduced genomes. | Bacteria of the CPR clade (such as *Vampirococcus lugosii*) and Archaea of the DPANN | (Castelle and Banfield 2018, Moreira et al 2000) |
| Viruses | Marine isolates support the “Cryptic Escape Theory”. In here small cell size is a strategy to minimize viral predation. This article also finds a correlation between genome size and cell size. | Diverse bacterial taxa (Cyanobacteria, Proteobacteria, Actinobacteria, among others) | (Yooseph et al 2010) |

**Table References**

Allen LZ, Allen EE, Badger JH, McCrow JP, Paulsen IT, Elbourne LD *et al* (2012). Influence of nutrients and currents on the genomic composition of microbes across an upwelling mosaic. *ISME J* **6:** 1403-1414 10.1038/ismej.2011.201.

Aylward FO, Santoro AE (2020). Heterotrophic Thaumarchaea with Small Genomes Are Widespread in the Dark Ocean. *mSystems* **5** 10.1128/mSystems.00415-20.

Berube PM, Biller SJ, Hackl T, Hogle SL, Satinsky BM, Becker JW *et al* (2018). Single cell genomes of Prochlorococcus, Synechococcus, and sympatric microbes from diverse marine environments. *Sci Data* **5:** 180154 10.1038/sdata.2018.154.

Blesa A, Averhoff B, Berenguer J (2018). Horizontal Gene Transfer in Thermus spp. *Curr Issues Mol Biol* **29:** 23-36 10.21775/cimb.029.023.

Borges KM, Bergquist PL (1993). Genomic restriction map of the extremely thermophilic bacterium Thermus thermophilus HB8. *J Bacteriol* **175:** 103-110 10.1128/jb.175.1.103-110.1993.

Boussau B, Karlberg EO, Frank AC, Legault BA, Andersson SG (2004). Computational inference of scenarios for alpha-proteobacterial genome evolution. *Proc Natl Acad Sci U S A* **101:** 9722-9727 10.1073/pnas.0400975101.

Brewer TE, Handley KM, Carini P, Gilbert JA, Fierer N (2016). Genome reduction in an abundant and ubiquitous soil bacterium ‘Candidatus Udaeobacter copiosus’. *Nature Microbiology* **2:** 16198 10.1038/nmicrobiol.2016.198.

Brochier-Armanet C, Deschamps P, Lopez-Garcia P, Zivanovic Y, Rodriguez-Valera F, Moreira D (2011). Complete-fosmid and fosmid-end sequences reveal frequent horizontal gene transfers in marine uncultured planktonic archaea. *ISME J* **5:** 1291-1302 10.1038/ismej.2011.16.

Bromfield ESP, Cloutier S, Nguyen HDT (2019). Description and complete genome sequence of Bradyrhizobium amphicarpaeae sp. nov., harbouring photosystem and nitrogen-fixation genes. *Int J Syst Evol Microbiol* **69:** 2841-2848 10.1099/ijsem.0.003569.

Cabello-Yeves PJ, Zemskaya TI, Rosselli R, Coutinho FH, Zakharenko AS, Blinov VV *et al* (2018). Genomes of Novel Microbial Lineages Assembled from the Sub-Ice Waters of Lake Baikal. *Appl Environ Microbiol* **84** 10.1128/AEM.02132-17.

Castelle CJ, Banfield JF (2018). Major New Microbial Groups Expand Diversity and Alter our Understanding of the Tree of Life. *Cell* **172:** 1181-1197 10.1016/j.cell.2018.02.016.

Chu X, Li S, Wang S, Luo D, Luo H (2020). Gene loss through pseudogenization contributes to the ecological diversification of a generalist Roseobacter lineage. *ISME J* **15:** 489-502 10.1038/s41396-020-00790-0.

de Souza RSC, Armanhi JSL, Damasceno NB, Imperial J, Arruda P (2019). Genome Sequences of a Plant Beneficial Synthetic Bacterial Community Reveal Genetic Features for Successful Plant Colonization. *Frontiers in microbiology* **10:** 1779 10.3389/fmicb.2019.01779.

Di Rienzi SC, Sharon I, Wrighton KC, Koren O, Hug LA, Thomas BC *et al* (2013). The human gut and groundwater harbor non-photosynthetic bacteria belonging to a new candidate phylum sibling to Cyanobacteria. *eLife* **2:** e01102 10.7554/eLife.01102.

Dieser M, Smith HJ, Ramaraj T, Foreman CM (2019). Janthinobacterium CG23_2: Comparative Genome Analysis Reveals Enhanced Environmental Sensing and Transcriptional Regulation for Adaptation to Life in an Antarctic Supraglacial Stream. *Microorganisms* **7** 10.3390/microorganisms7100454.

Dufresne A, Salanoubat M, Partensky F, Artiguenave F, Axmann IM, Barbe V *et al* (2003). Genome sequence of the cyanobacterium Prochlorococcus marinus SS120, a nearly minimal oxyphototrophic genome. *Proc Natl Acad Sci U S A* **100:** 10020-10025 10.1073/pnas.1733211100.

Gagunashvili AN, Andresson OS (2018). Distinctive characters of Nostoc genomes in cyanolichens. *BMC genomics* **19:** 434 10.1186/s12864-018-4743-5.

Garcia SL, Buck M, McMahon KD, Grossart HP, Eiler A, Warnecke F (2015). Auxotrophy and intrapopulation complementary in the "interactome' of a cultivated freshwater model community. *Molecular ecology* **24:** 4449-4459 10.1111/mec.13319.

Grote J, Thrash JC, Huggett MJ, Landry ZC, Carini P, Giovannoni SJ *et al* (2012). Streamlining and Core Genome Conservation among Highly Divergent Members of the SAR11 Clade. *Mbio* **3** 10.1128/mBio.00252-12.

Kang I, Kim S, Islam MR, Cho J-C (2017). The first complete genome sequences of the acI lineage, the most abundant freshwater Actinobacteria, obtained by whole-genome-amplification of dilution-to-extinction cultures. *Scientific reports* **7** 10.1038/srep42252.

Konstantinidis KT, Tiedje JM (2004). Trends between gene content and genome size in prokaryotic species with larger genomes. *Proc Natl Acad Sci U S A* **101:** 3160-3165 10.1073/pnas.0308653100.

Lauro FM, McDougald D, Thomas T, Williams TJ, Egan S, Rice S *et al* (2009). The genomic basis of trophic strategy in marine bacteria. *Proc Natl Acad Sci U S A* **106:** 15527-15533 10.1073/pnas.0903507106.

Levy A, Salas Gonzalez I, Mittelviefhaus M, Clingenpeel S, Herrera Paredes S, Miao J *et al* (2017). Genomic features of bacterial adaptation to plants. *Nat Genet* **50:** 138-150 10.1038/s41588-017-0012-9.

Makarova K, Slesarev A, Wolf Y, Sorokin A, Mirkin B, Koonin E *et al* (2006). Comparative genomics of the lactic acid bacteria. *Proc Natl Acad Sci U S A* **103:** 15611-15616 10.1073/pnas.0607117103.

McLean JS, Bor B, Kerns KA, Liu Q, To TT, Solden L *et al* (2020). Acquisition and Adaptation of Ultra-small Parasitic Reduced Genome Bacteria to Mammalian Hosts. *Cell reports* **32:** 107939 10.1016/j.celrep.2020.107939.

Mende DR, Bryant JA, Aylward FO, Eppley JM, Nielsen T, Karl DM *et al* (2017). Environmental drivers of a microbial genomic transition zone in the ocean's interior. *Nat Microbiol* **2:** 1367-1373 10.1038/s41564-017-0008-3.

Moreira D, Le Guyader H, Philippe H (2000). The origin of red algae and the evolution of chloroplasts. *Nature* **405:** 69-72 Doi 10.1038/35011054.

Nielsen DA, Fierer N, Geoghegan JL, Gillings MR, Gumerov V, Madin JS *et al* (2021). Aerobic bacteria and archaea tend to have larger and more versatile genomes. *Oikos* **130:** 501-511 10.1111/oik.07912.

Nilsson AI, Koskiniemi S, Eriksson S, Kugelberg E, Hinton JC, Andersson DI (2005). Bacterial genome size reduction by experimental evolution. *Proc Natl Acad Sci U S A* **102:** 12112-12116 10.1073/pnas.0503654102.

Okubo T, Fukushima S, Itakura M, Oshima K, Longtonglang A, Teaumroong N *et al* (2013). Genome analysis suggests that the soil oligotrophic bacterium Agromonas oligotrophica (Bradyrhizobium oligotrophicum) is a nitrogen-fixing symbiont of Aeschynomene indica. *Appl Environ Microbiol* **79:** 2542-2551 10.1128/AEM.00009-13.

Rhodes ME, Spear JR, Oren A, House CH (2011). Differences in lateral gene transfer in hypersaline versus thermal environments. *BMC evolutionary biology* **11:** 199 10.1186/1471-2148-11-199.

Rocap G, Larimer FW, Lamerdin J, Malfatti S, Chain P, Ahlgren NA *et al* (2003). Genome divergence in two Prochlorococcus ecotypes reflects oceanic niche differentiation. *Nature* **424:** 1042-1047 10.1038/nature01947.

Saifuddin M, Bhatnagar JM, Segre D, Finzi AC (2019). Microbial carbon use efficiency predicted from genome-scale metabolic models. *Nat Commun* **10:** 3568 10.1038/s41467-019-11488-z.

Smith MW, Zeigler Allen L, Allen AE, Herfort L, Simon HM (2013). Contrasting genomic properties of free-living and particle-attached microbial assemblages within a coastal ecosystem. *Frontiers in microbiology* **4:** 120 10.3389/fmicb.2013.00120.

Sorensen JW, Dunivin TK, Tobin TC, Shade A (2019). Ecological selection for small microbial genomes along a temperate-to-thermal soil gradient. *Nat Microbiol* **4:** 55-61 10.1038/s41564-018-0276-6.

Tamas I, Klasson L, Canback B, Naslund AK, Eriksson AS, Wernegreen JJ *et al* (2002). 50 million years of genomic stasis in endosymbiotic bacteria. *Science* **296:** 2376-2379 10.1126/science.1071278.

Yooseph S, Nealson KH, Rusch DB, McCrow JP, Dupont CL, Kim M *et al* (2010). Genomic and functional adaptation in surface ocean planktonic prokaryotes. *Nature* **468:** 60-66 10.1038/nature09530.
